# Impact of knowledge transfer through the implementation of a telemedicine program in a community hospital in Brazil

**DOI:** 10.1186/cc12463

**Published:** 2013-03-19

**Authors:** CA Abreu Filho, M Steinman, A Andrade, R Cal, N Akamine, J Teixeira, E Silva, A Kanamura, M Cenderoglo, C Lottenberg

**Affiliations:** 1 Hospital Israelita Albert Einstein, São Paulo, Brazil; 2 Hospital Municipal Dr. Moysés Deutsch, São Paulo, Brazil

## Introduction

Emergency survival rates vary significantly according to the quality of care, which depends on human and technological resources. Emergency and critical care medicine physicians must make fast decisions; the presence of experienced consultants improves survival. In developing countries, there is a shortage of skilled doctors. The aim is to describe the first Brazilian initiative of real-time teleconferencing telemedicine (TM) providing 24/7 emergency department (ED) and ICU coverage.

## Methods

Since May 2012 a TM program has been implemented at two hospitals in São Paulo, Brazil - Hospital Municipal Dr. Moysés Deutsch (HMMD), a public, secondary hospital, and Hospital Israelita Albert Einstein (HIAE), a tertiary private philanthropic entity - due to a partnership with the Brazilian Health Ministry. TM Central Command was located at HIAE with Endpoint 97 MXP Cisco^® ^Solution and a mobile Intern MXP ISDN/IP Cisco^® ^for the remote hospital (HMMD) via dedicated GB/sec connection. Imaging examinations were evaluated using PACS technology. Every recruited patient was assessed by the Central Command through TM with an experienced consultant.

## Results

Over a 6-month period, 131 teleconsultations (114 patients) were done. Mean age was 50.1 years, 57.1% was male and mean APACHE II score was 24.3. A total of 64.8% originated from the ICU and 35.2% from the ED. Main consultation diagnoses were sepsis (31.3%); stroke (29.8%); survival from cardiac arrest (6.1%); trauma (6.1%); and acute myocardial infarction (5.3%). TM improved diagnosis in 14.5% and influenced the clinical management in 85.5% of the consultations. Invasive procedures were indicated in 61.1%. Life-saving procedures were TM related in seven patients (6.1%): stroke thrombolysis (*n *= 6) and limb amputation (*n *= 1). Seven patients (6.1%) were transferred and submitted to surgical procedures (heart surgery (*n *= 2), neurosurgery (*n *= 4) and liver transplantation (*n *= 1)). The majority of the patients remained at HMMD and were discharged.

## Conclusion

A TM program is feasible to be implemented in a community hospital. The major benefit is expertise medical transfer from the tertiary hospital to the community setting, improving diagnosis and management of critical care patients, and avoiding routine transfer to a major urban center.

